# Supplementary material for: De Novo Transcriptome Assembly and Analysis of Longevity Genes Using Subterranean Termite (Reticulitermes chinensis) Castes
Source: Int J Mol Sci. 2022 Nov 7;23(21):13660. doi: 10.3390/ijms232113660 (PMC9657995; doi:10.3390/ijms232113660)
Supplement: Supplementary file 1 [file ijms-23-13660-s001.zip › ijms-1877527-supplementary.pdf]

International Journal of *Molecular Sciences*
MDPI

Supplementary Materials

De Novo Transcriptome Assembly and Analysis of Longevity Genes Using Subterranean Termite (*Reticulitermes chinensis*) Castes

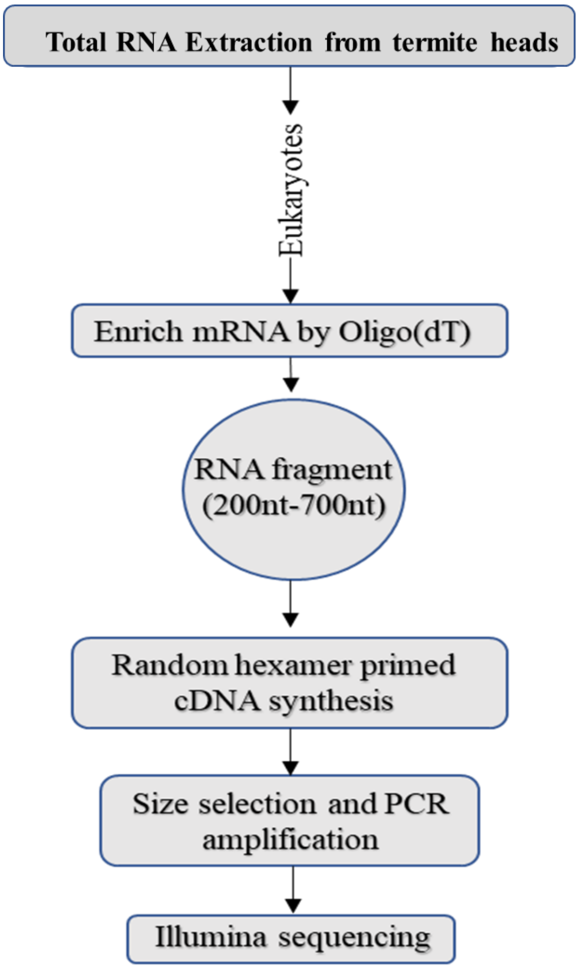

Figure S1. The procedure of total RNA extraction and Illumina sequencing.

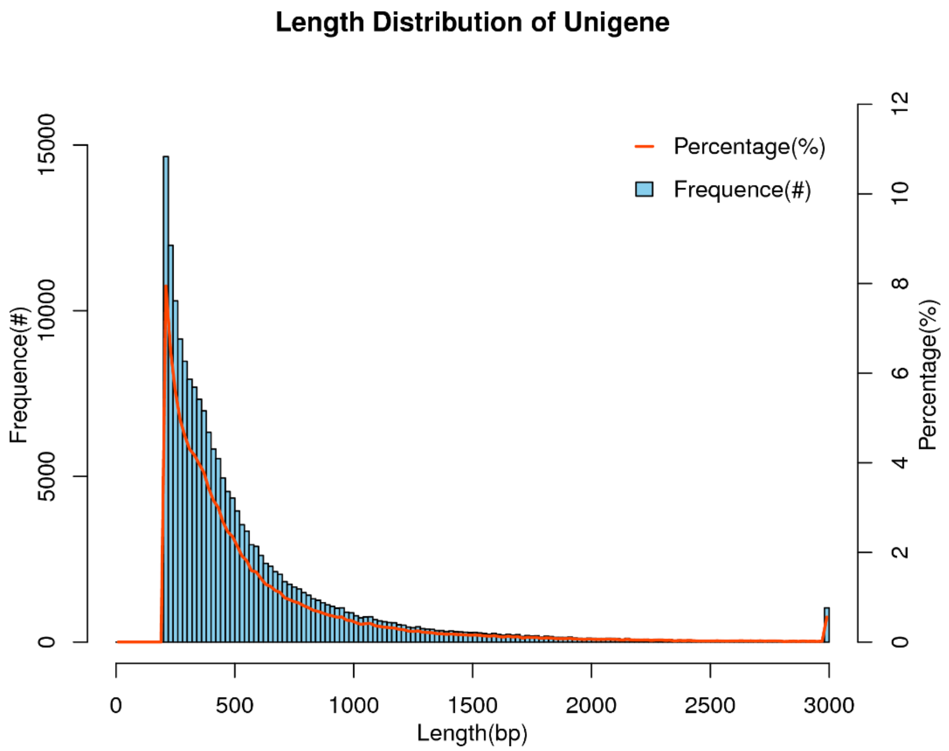

Figure S2. The length distribution of unigenes of the *R. chinensis*. The histogram represents the total assembled unigenes and length distribution for the identified significant matches. The x-axis indicates the sequence length (bp) sizes from >200 nt to >3000 nt. The left side of the y-axis shows the frequency (♯) (blue) and the right side of the y-axis indicates the percentage (%) (red) of unigenes for every given size. The results of the sequence-length matches (with a cut-off E-value of 1.0E-5) in the Nr databases were higher among the longer assembled sequences.

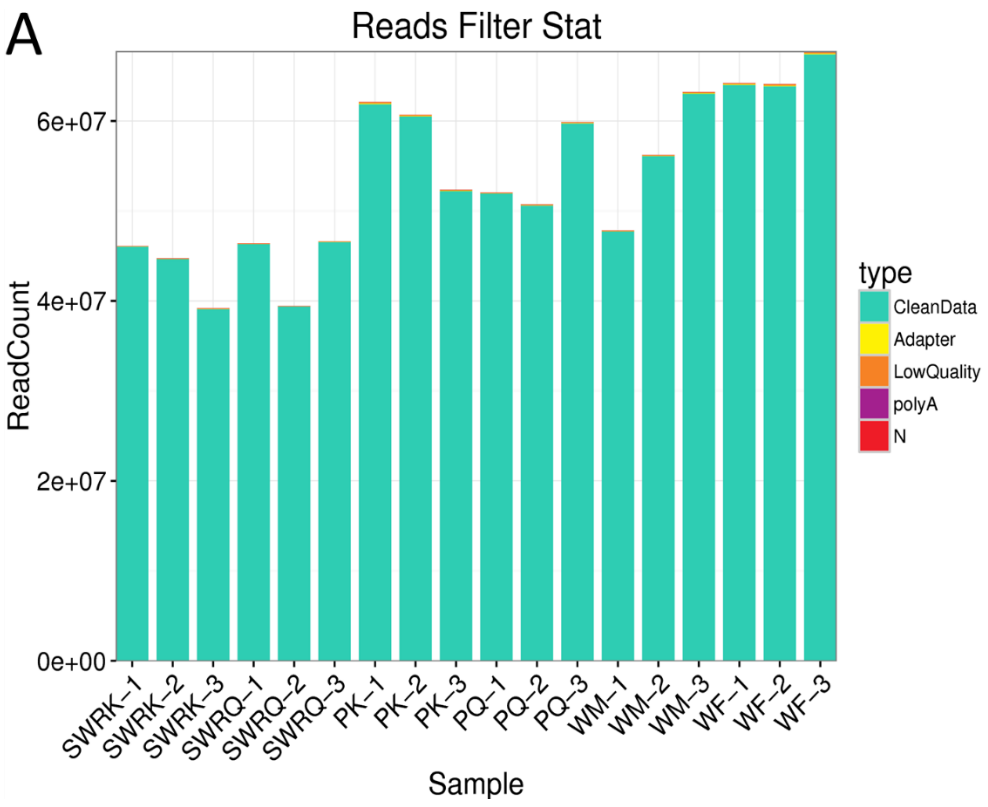

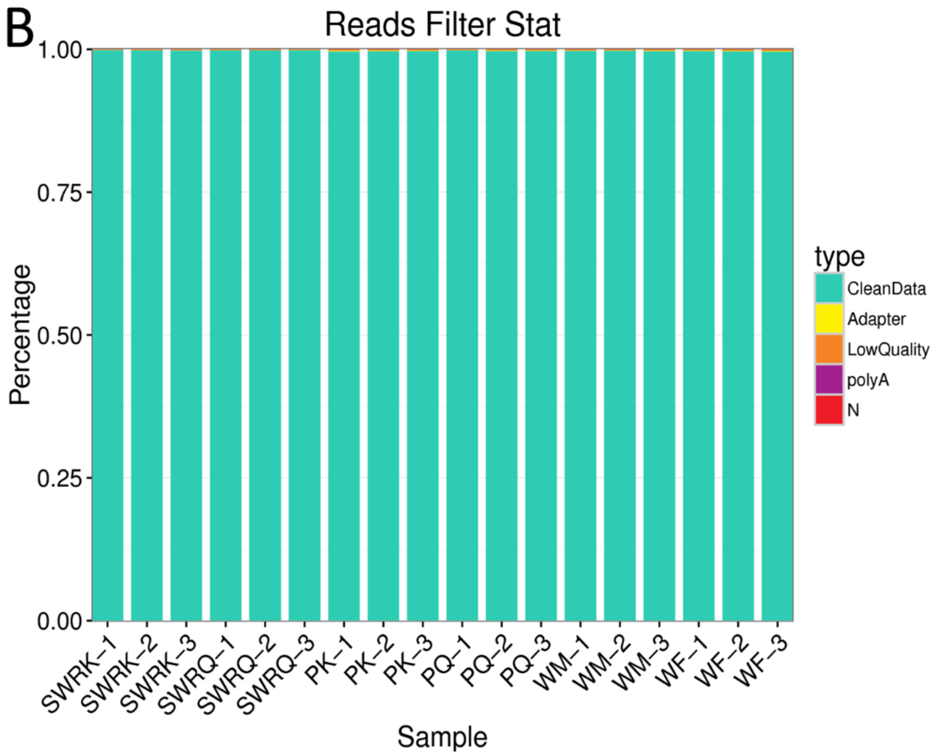

Figure S3. The filtering statistics chart is as follows: the (**A**) picture is based on the value of the filtering statistic, and (**B**) picture is based on the filtering statistics percentage.

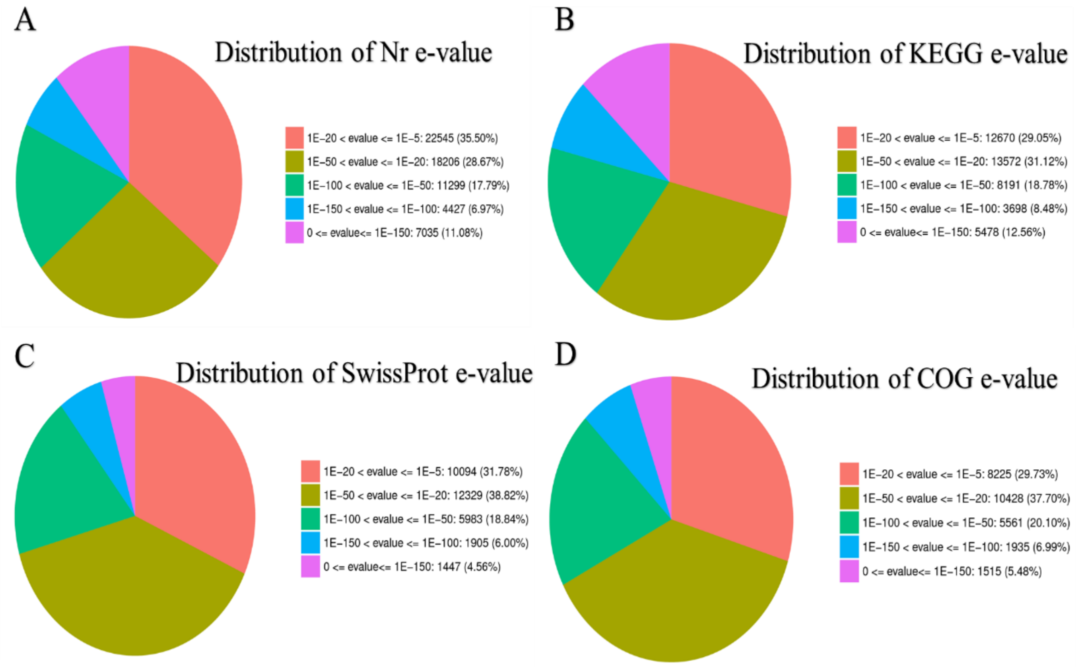

Figure S4. Distribution of e-value in Nr, KEGG, SwissProt, and COG. Each color indicates different E-values.

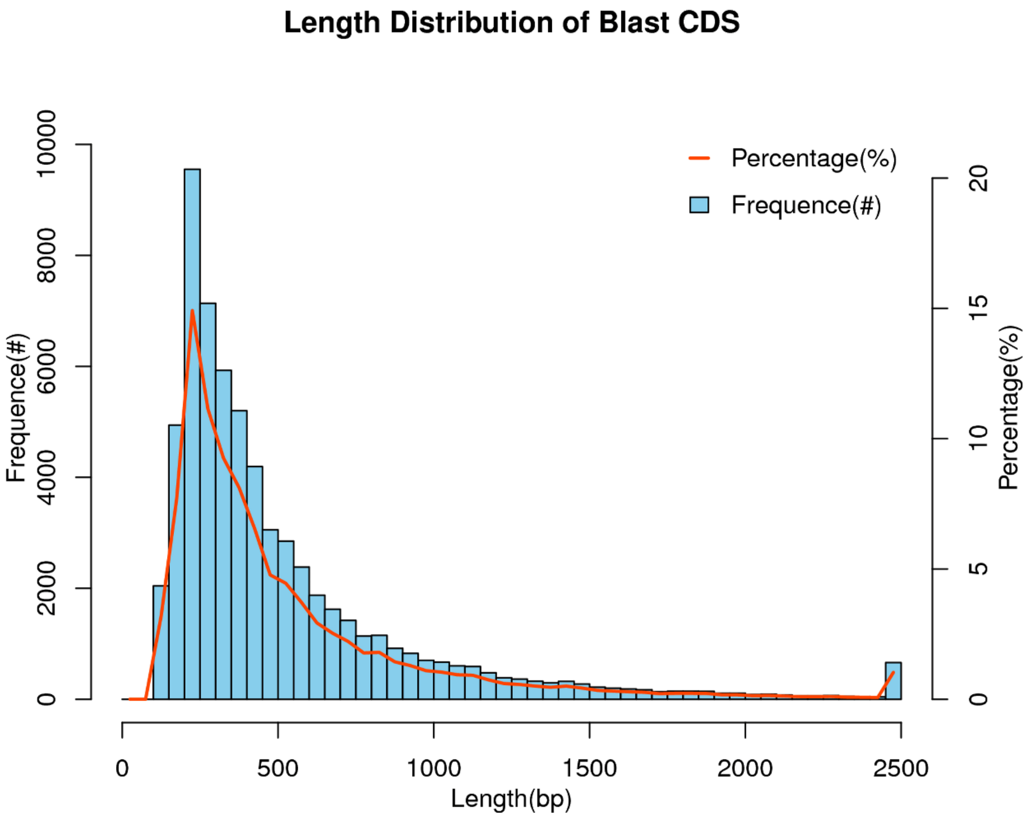

Figure S5. The length distribution of Protein-Coding Region prediction (CDS) from BLAST. A total of 64,303 predicted unigenes using BLASTX histogram indicate the sequence-length distribution for significant matches that were found. The x-axis shows the sequence length (bp) size from 0 nt to >3000 nt. The left side of the y-axis indicates the frequency (♯) (blue), and the right side of the y-axis shows the percentage (%) (red) of unigenes for every given size.

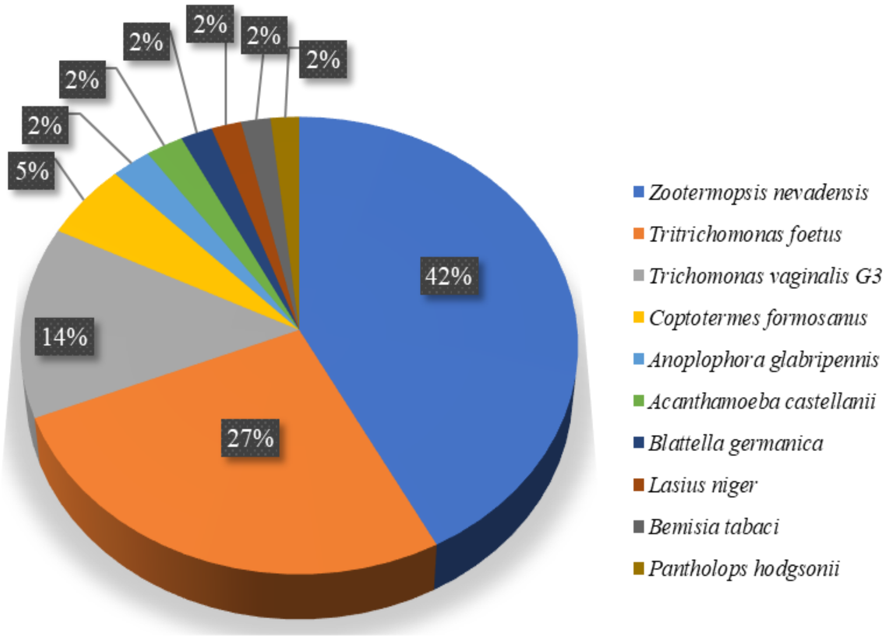

Figure S6. BLASTX results for species distribution. The Nr database sequence with the lowest value (E) of each unigene in the Nr is the corresponding homologous sequence by using blastx to compare the assembled unigenes sequences with the Nr database. The different colors represent various species.

Table S1. The six selected genes from ***R. chinensis*** and their primers were used in RT-qPCR analyses.

Gene ID	Symbol	Primers Sequences
	Beta-actin	Forward: CCCAACACAGCGTCTTACAA<br>Reverse: CAGATGTCCTCAGCTTCACG
Unigene 0082575	*PdK1*	Forward: TCCTCCTCCTGCTACTGCTGAAG<br>Reverse: CGACATATGACGGAGTAGGTGGTG
Unigene 0034890	*akt2-a*	Forward: CCAAGAAGTATGTCGAAAGAAGTCA<br>Reverse: TCTGTGAAACCATCTCCCAATTAAG
Unigene 0092210	*Tsc2*	Forward: AGTGGTGCTAACATGCCTGC<br>Reverse: ACCTTCCAGCTGCTCTGACA
Unigene 0063105	*mTOR*	Forward: GGCTTGAAGGGTGTTCCACA<br>Reverse: GCTTACCTGTAGGCGGCAAT
Unigene 0011832	*EIF4E*	Forward: GGATCTCGTCTTGGCCGTCATTG<br>Reverse: AGCAACCTCGTGAGCCACTCC
Unigene 0155613	*RPS6*	Forward: TCTATGACAAGCGCCTTGGAGAAG<br>Reverse: CTTGGACGGAGGCAGGTGGATC

The complete sequence of the unigenes 0082575; 0034890; 0092210; 0063105; 0011832 and 0155613 for the primers design.

>Unigene0082575 + nr
ACCAGAAGTGTTAAATAACGGAAAGATTTCGCCTGCAAGTGATCTCTGGAGCTTCGGTTG
TATTTTGTTTTCCATTATCGACGGAAAGCCTCCCTTTTATACAGGGAACATGATGAATAC
ATTTGCTGCTATCGAGAAAGGAACCATTGACTTTCCACCGACTTTCTTGCCACAAGCGAA
AGACCTTGTTCAGAAACTTTTGAAGCTCGAACCCTCTGAACGATTGGGTTTCAATGAGTA
CCCGACCAATTACAAGCCCATTCGAGACCATCCATTCTTTTCTGGATTGGATTGGGAGAC
TCTTCCACTTGAAGAAGTTCCTCCTTTCGATGACGATGAACCATTTATCCTTCCTCCTCC
TGCTACTGCTGAAGTACCTTCATCCAAACCTTCTTCGAAACCTCCTCCTTCTGATTCTCC
TCCAGATAGTTCTCCCTCTCCGAAAGCCTCTTCACCAGCTCCTTCTTCTCCCGCTCCTGC
ACCACCTACTCCGTCATATGTCGTTGATCCTGCTAAGTATCTCAGTCTCATCAATTCTCC
TAACCTGCTTGATGAATCAAAACAATTTCTGATTCAAGACGAGGTAATTATCTATCAAGG
GCTTCTGTGGAAGCGTGTTGGACTTTCAAATAAGGAAAGGTTGTTTGTGATAACGACAAG
GCCTCGAATCTTCTACTGGGACTTGAAGGGCAAAAAATTCAAAGGAGAGATTCCTCTTTC
AAAAGAACTTGAGGTTTCCATTGAAAAAGGAGGAAAGTTTATTCTCGAAGTGCCCGGCCG
TGTGTATAAGCTCG
>Unigene0034890 + nr
GCCTTTTTACTGCGAAAACCAGAATCAAATGTTCAAAGATATCCAATCAAAGCAAGTCAA
GTATCCAAGAAGTATGTCGAAAGAAGTCAAAGACTTGATTTCGAGATTTCTTGTCCGTGA
CATCTCTCAGAGAATTGGAGCTGGTCCAGAAGATTATGAAGAAGTTAAGAGACATCCTTG
GTTTAGTGATCTTAATTGGGAGATGGTTTCACAGAAAAAGATTACACCTGAATGGAAGCC
TG
>Unigene0092210 275 1060 Tuberin [Zootermopsis nevadensis]
ATGAGTGCGAAGGATAAGGAAAACAAAACTTTTCACGAGAAACTGAAGCAGTTTTTCAGA
ATAAACAAAGGTGGAACAGGTAACTTGAAAGGCAGAGTGGATTTTGCTTTGACTCAAGAC
ATTGAGAAAGACCTCAGCCCTGAAAACCCAGTGACTCATCGCGTGAAGGTTATAAAGGAA
CTGAGTGAAGCCGTTCTTAAGAACCGTCTTGAGGATAATTCAATTGAGAAATTATGGGCA
TGTCTTCAAGATTTGTTACATCGTGAAGTACTAAAAGAACATCGCCATCTTGCTTTTTAC
TTTTTCCGTTGCCTTGTACAGGGACAGTATGACAAACTAGGGCTTATGAGGGTTCACTTC
TTCAGAATCATTAAGACTCATGATATTCCAGAAGATGTAGCACCAAGATTTGAGCTGTTG
CAAAGTCTGACAGACAACGGGAAAGACATTCTGTACTTTGAAGAAGAGGTGGGGCCTTTT
CTACTTTACTGGATGCCAGCTGTGATTGGTGTTGCTCGCACAAAGGAATTCCTCTCCATG
CTTGTCAATGTTATCAAGTTTAATGCTGCTTATGTTGATGAGGATGTTATTTCAGGTCTT
GTTCAGAATACATGTTTCCTGTGTTGTTGGAGCAACTCGGAGGAAGTGGTGCTAACATGC
CTGCAAGTGCTGGACACAGTTGTCTGTTACAGTACCTTGCCTTCTGACTCTCTGCATACC
TTTATCAGTGCCCTTTGTCGAACTGTCAATGTAGAAGCGTATTGTCAGAGCAGCTGGAAG

GTGATG
>Unigene0063105 3 665 PIKK family atypical protein kinase [Trichomonas vaginalis G3]
AATCAAGAGTCTCTTCATCAAGCTTGGCAGTTCTATATTAATCTATATCGTCAAGTGAAA
ATGATTGTTCTAAACTTAATGACAATTCCTCTTGCTGAAGCTTCTCCTAAACTGTCCTCA
GTTTTAAGCTTTTCTTTGTCTGTCCCTGGAACTTACCATCACAATAGTAATATCATTACG
ATTCAATCATTTCAACCTCTTTTAAAAGTTTTACCTTCGAAGCAAAGACCCAGAAGAATG
GGAATTATTGGAAGCGATGGAAATTCTTATACATTTTTGTTGAAAGCAAGAGAAGACACT
CGTCTTGATGAGCGAGTTATGCAGTTATTCACTTTCTTAACTTCACTTGTGAACAGTTCA
GCAATTCCAATGAAAAACAAGTTAACGATTACAACTTATAATGTCATTCCTTTAACACAT
GAAGTTGGATTAATTGGTTGGCTTGAAGGGTGTTCCACAATTTATGATCTTATCTTGGAA
CATCGAAAGAAAAATTCAATCGCAACTAAAAAGGAATATGAATATGCAATTAAAAAATAT
CCAACTTATAATCAATTGCCGCCTACAGGTAAGCTCAAGGCATTCAGAGAATCCTTAAAT
GAAACCAAAGGAGATGATTTAAAACAATTTCTCTTCAAGTTTTCAACAGATTCCTCTAAT
TGG
>Unigene0082575 + nr
ACCAGAAGTGTTAAATAACGGAAAGATTTCGCCTGCAAGTGATCTCTGGAGCTTCGGTTG
TATTTTGTTTTCCATTATCGACGGAAAGCCTCCCTTTTATACAGGGAACATGATGAATAC
ATTTGCTGCTATCGAGAAAGGAACCATTGACTTTCCACCGACTTTCTTGCCACAAGCGAA
AGACCTTGTTCAGAAACTTTTGAAGCTCGAACCCTCTGAACGATTGGGTTTCAATGAGTA
CCCGACCAATTACAAGCCCATTCGAGACCATCCATTCTTTTCTGGATTGGATTGGGAGAC
TCTTCCACTTGAAGAAGTTCCTCCTTTCGATGACGATGAACCATTTATCCTTCCTCCTCC
TGCTACTGCTGAAGTACCTTCATCCAAACCTTCTTCGAAACCTCCTCCTTCTGATTCTCC
TCCAGATAGTTCTCCCTCTCCGAAAGCCTCTTCACCAGCTCCTTCTTCTCCCGCTCCTGC
ACCACCTACTCCGTCATATGTCGTTGATCCTGCTAAGTATCTCAGTCTCATCAATTCTCC
TAACCTGCTTGATGAATCAAAACAATTTCTGATTCAAGACGAGGTAATTATCTATCAAGG
GCTTCTGTGGAAGCGTGTTGGACTTTCAAATAAGGAAAGGTTGTTTGTGATAACGACAAG
GCCTCGAATCTTCTACTGGGACTTGAAGGGCAAAAAATTCAAAGGAGAGATTCCTCTTTC
AAAAGAACTTGAGGTTTCCATTGAAAAAGGAGGAAAGTTTATTCTCGAAGTGCCCGGCCG
TGTGTATAAGCTCG
>Unigene0155613 - nr
CCGAAAACGGGACACAGATCTGCGTCGAAGTTACTGAACAGCAGCAGCTCGCGAACCTCT
ATGACAAGCGCCTTGGAGAAGATATTGAAGGAGAGAAGCTCGGGGATCAATTCGCAGGTT
ATGTTTTTCGCCTTGGTGGAGGCTTTGACAAGGAAGGTTTTCCAATGAAACCAGGTGTTT
TCACTCCTCGTCGAGTTCGTCTTCTCTTAAAAAAGGGATCCACCTGCTTCCGTCCAAGAG
TTAACGGAGAAAGGAAAAGAAAGTCTGTTCGTGGCTGTATCATTTCTTCTGAGATTTCCG
CGCTTCACATTATTGTCATTCAGAAAGGTCCTGGAGAGATTCCAGGTCTCACTGATCTCC
ATGTCCCAAGATTGTATAGCCCCAAGCGTGCTTCCACACTGAAGAAGATGTTTGATCTTA
AGACAAATGAAGAGGTAGCTAATGCAGCTATTCAAAGGCAGACTAAGTCAGGTAGGTTTG
TGAAACCAAAGATCCAGAGACTAATTACTCCTCGCAGACTTCAACGAAAGAAGAAAGAAC
AACAAGAAAG

Table S2. Data quality control and low-quality data filtering.

Sample	RawDatas	CleanData (%)	Adapter (%)	LowQuality (%)	polyA (%)	N(%)
SWRK-1	46103502	46038870 (99.86%)	13658 (0.03%)	49558 (0.11%)	0 (0.00%)	1416 (0.00%)
SWRK-2	44745990	44657610 (99.80%)	18150 (0.04%)	68768 (0.15%)	0 (0.00%)	1462 (0.00%)
SWRK-3	39179304	39095042 (99.78%)	15522 (0.04%)	67192 (0.17%)	0 (0.00%)	1548 (0.00%)
SWRQ-1	46408498	46331166 (99.83%)	15840 (0.03%)	60006 (0.13%)	0 (0.00%)	1486 (0.00%)
SWRQ-2	39446624	39376280 (99.82%)	12208 (0.03%)	56544 (0.14%)	0 (0.00%)	1592 (0.00%)
SWRQ-3	46614010	46545376 (99.85%)	12572 (0.03%)	54548 (0.12%)	0 (0.00%)	1514 (0.00%)
PK-1	62105464	61875302 (99.63%)	85748 (0.14%)	141472 (0.23%)	0 (0.00%)	2942 (0.00%)
PK-2	60692886	60491486 (99.67%)	71746 (0.12%)	126622 (0.21%)	0 (0.00%)	3032 (0.00%)
PK-3	52364296	52211218 (99.71%)	44148 (0.08%)	106584 (0.20%)	0 (0.00%)	2346 (0.00%)
PQ-1	52043604	51946682 (99.81%)	22858 (0.04%)	73272 (0.14%)	0 (0.00%)	792 (0.00%)
PQ-2	50747682	50594180 (99.70%)	44976 (0.09%)	107766 (0.21%)	0 (0.00%)	760 (0.00%)
PQ-3	59891698	59718954 (99.71%)	49612 (0.08%)	122380 (0.20%)	0 (0.00%)	752 (0.00%)
WM-1	47844384	47709742 (99.72%)	31952 (0.07%)	102690 (0.21%)	0 (0.00%)	0 (0.00%)
WM-2	56230146	56089922 (99.75%)	39216 (0.07%)	98568 (0.18%)	0 (0.00%)	2440 (0.00%)
WM-3	63230150	63032988 (99.69%)	63292 (0.10%)	131246 (0.21%)	0 (0.00%)	2624 (0.00%)
WF-1	64211886	64019360 (99.70%)	57852 (0.09%)	131876 (0.21%)	0 (0.00%)	2798 (0.00%)
WF-2	64091688	63868690 (99.65%)	70640 (0.11%)	149580 (0.23%)	0 (0.00%)	2778 (0.00%)
WF-3	67680970	67410876 (99.60%)	97420 (0.14%)	169744 (0.25%)	0 (0.00%)	2930 (0.00%)
